# Chronic stress impairs GABAergic control of amygdala through suppressing the tonic GABAA receptor currents

**DOI:** 10.1186/1756-6606-7-32

**Published:** 2014-04-24

**Authors:** Zhi-Peng Liu, Chen Song, Min Wang, Ye He, Xiao-Bin Xu, Han-Qing Pan, Wen-Bing Chen, Wei-Jie Peng, Bing-Xing Pan

**Affiliations:** 1 Laboratory of Fear and Anxiety Disorders, Institute of Life Science, Nanchang University, Nanchang 330031, China; 2 Department of Pharmacology, Nanchang University, Nanchang 330031, China

**Keywords:** Amygdala, Chronic stress, GABA, Electrophysiology, Tonic inhibition, Corticosterone, Glucocorticoid receptor, Neuronal excitability

## Abstract

**Background:**

Chronic stress is generally known to exacerbate the development of numerous neuropsychiatric diseases such as fear and anxiety disorders, which is at least partially due to the disinhibition of amygdala subsequent to the prolonged stress exposure. GABA receptor A (GABA_A_R) mediates the primary component of inhibition in brain and its activation produces two forms of inhibition: the phasic and tonic inhibition. While both of them are critically engaged in limiting the activity of amygdala, their roles in the amygdala disinhibition subsequent to chronic stress exposure are largely unknown.

**Results:**

We investigated the possible alterations of phasic and tonic GABA_A_R currents and their roles in the amygdala disinhibition subsequent to chronic stress. We found that both chronic immobilization and unpredictable stress led to long lasting loss of tonic GABA_A_R currents in the projection neurons of lateral amygdala. By contrast, the phasic GABA_A_R currents, as measured by the spontaneous inhibitory postsynaptic currents, were virtually unaltered. The loss of tonic inhibition varied with the duration of daily stress and the total days of stress exposure. It was prevented by pretreatment with metyrapone to block corticosterone synthesis or RU 38486, a glucocorticoid receptor antagonist, suggesting the critical involvement of glucocorticoid receptor activation. Moreover, chronic treatment with corticosterone mimicked the effect of chronic stress and reduced the tonic inhibition in lateral amygdala of control mice. The loss of tonic inhibition resulted in the impaired GABAergic gating on neuronal excitability in amygdala, which was prevented by metyrapone pretreatment.

**Conclusions:**

Our study suggests that enduring loss of tonic but not phasic GABA_A_R currents critically contributes to the prolonged amygdala disinhibition subsequent to chronic stress. We propose that the preferential loss of tonic inhibition may account for the development of stress-related neuropsychiatric diseases.

## Background

Repeated exposure to stress has enduring detrimental influence on the brain and body function [1]. It enhances the sufferers’ reactivity to the environmentally threatening or emotionally challenging events and at the extreme, leads to the development of a series of mental disorders including anxiety disorders and depression [2,3]. Amygdala, an almond-shape brain nucleus complex located deep within the temporal lobe, is critically engaged in the acquisition, retrieval and expression of aversive memories [4-6]. Mounting evidence has demonstrated that amygdala is one of the primary targets of chronic stress [7,8]. Under resting conditions, the amygdala is inhibited by the extensive GABAergic network and exhibits low neuronal firing [9]. By contrast, the amygdala is disinhibited and shows heightened activation upon chronic stress [10,11], resulting in the increased sensitivity of amygdala to the environmental cues and individual’s hypervigilance which persist even after long period of recovery.

Studies on the neuronal and molecular underpinnings of chronic stress-induced amygdala hyperresponsiveness have revealed the involvement of multiple factors such as the structural remodeling of amygdala neurons inducing the dendritic arborization and spine hypertrophy [12,13] and the decreased expression of calcium-activated potassium channel (K_Ca_) in the cytoplasmic membrane [14]. The enlarged pools of spines facilitate amygdala neurons to receive and integrate the incoming signals from thalamocortical sensory domains and from the higher order cortical areas such as prefrontal cortex. The reduction of K_Ca_ activity, on the other hand, enhances the neuronal intrinsic excitability, thereby contributing to the overexcitation of amygdala as a consequence of chronic stress.

Besides these, chronic stress exposure was also reported to result in the attenuation of GABAergic signaling, shifting the amygdala to a more excitable state [9,15,16]. GABA_A_Rs mediate the majority of the inhibitory tone in central nervous system and their activation produces two forms of inhibition, the phasic and tonic inhibition [17,18]. They coexist in numerous brain areas and are thought to be mediated by intra- and extrasynaptic GABA_A_Rs respectively [19]. In amygdala, both forms of inhibition are engaged in constraining the neuronal activity [20,21]. However, their specific roles in amygdala disinhibition subsequent to chronic stress are largely unknown.

In this study, we investigated the possible effects of chronic immobilization and unpredictable stress on the phasic versus tonic GABA_A_R currents in mice LA with effort to examine their roles in subsequent amygdala disinhibition. Since the adverse effects of chronic stress always persist even upon the cessation of stress exposure [13], we conducted the study in mice experiencing 10 or 30 days of stress-free recovery from chronic immobilization or unpredictable stress exposure to explore the possible enduring alterations of GABA_A_R signaling. We found that chronic stress exposure led to long lasting loss of tonic but not phasic GABA_A_R currents through corticosterone (CORT) production with subsequent activation of glucocorticoid receptor (GR). The loss of tonic inhibition contributed substantially to the disinhibition of neuronal activity in LA following prolonged stress exposure.

## Results

### Chronic stress causes long lasting loss of tonic but not phasic GABA_A_R currents in LA PNs

**Figure 1:**
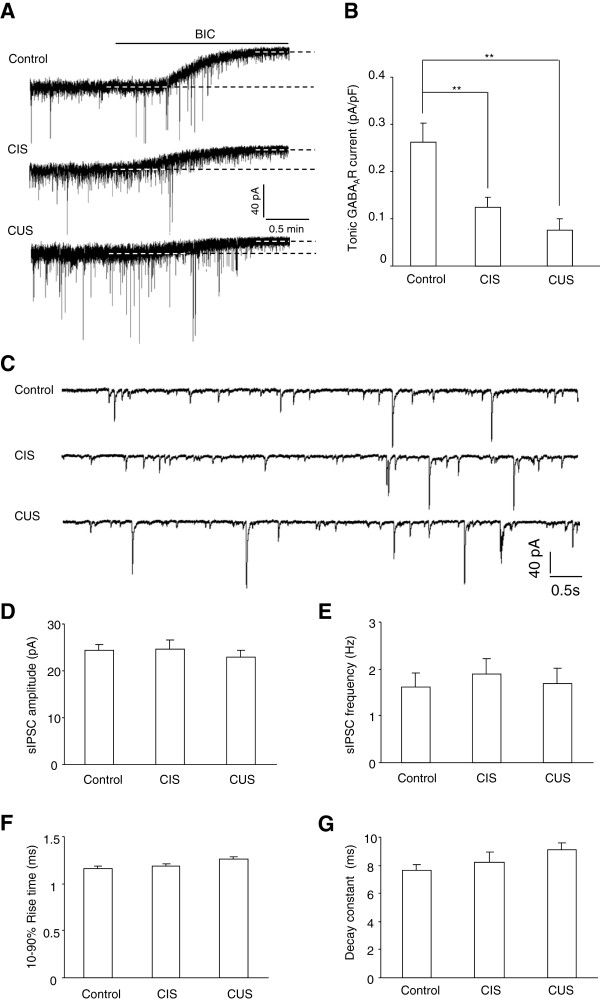
**Both chronic immobilization and unpredictable stress cause long lasting loss of tonic but not phasic inhibition in lateral amygdala. A**, Representative traces showing the tonic GABA_A_R currents in the projection neurons (PNs) of lateral amygdala (LA) from control mice (upper) and mice experiencing 10 days of recovery from chronic immobilization (CIS, middle) or unpredictable stress (CUS, bottom). The tonic currents were measured as the amplitude difference between the dashed lines indicating the average holding current before and after BIC application. **B**, Summary of the tonic GABA_A_R currents in control, CIS and CUS mice. **C**, Representative traces showing the spontaneous inhibitory postsynaptic currents (sIPSCs) in LA PNs from control (upper), CIS (middle) and CUS (bottom) mice. **D**-**G**, Summary of the sIPSCs amplitude **(D)**, frequency **(E)**, 10-90% rise time **(F)** and decay constant **(G)** in LA PNs from control, CIS and CUS mice. ** p < 0.01 (unpaired *t* test).

**Figure 2:**
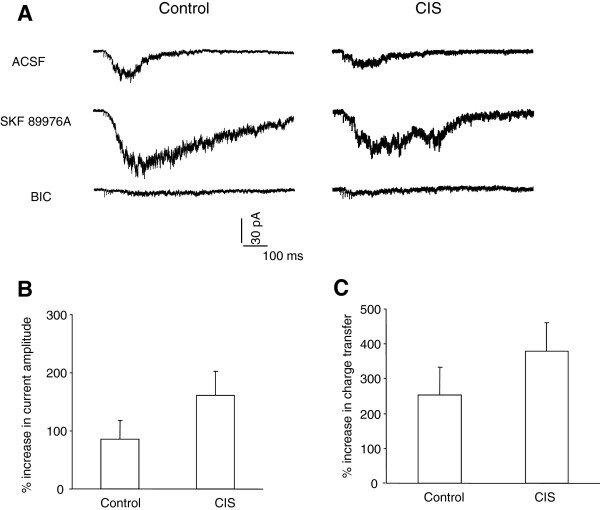
**The ambient GABA is similar in LA from control and CIS mice. A**, Representative traces showing GABA_A_R currents in an outside-out patch evoked by a short-lasting tetanus applied to the LA of control (left) and CIS mice (right). Slices were consecutively perfused with ACSF (top), SKF 89976A (middle) and BIC (bottom). Note that SKF 89976A enhanced the GABA_A_R currents in both mice. The stimulus artifacts were truncated for clarity. **B**-**C**, Comparisons of the increased amplitude **(B)** and charge transfer **(C)** of GABA_A_R currents by SKF 89976A in control and CIS mice.

Tonic GABAergic inhibition is generally known to result from the opening of peri- or extrasynaptic GABA_A_R upon binding the ambient GABA diffused outside of synapses. To investigate whether chronic stress had long lasting influence on the phasic versus tonic GABA_A_R currents in LA PNs, we recorded both currents from control mice and CIS mice having 10 days of recovery from stress (Figure 1). We found that the tonic GABA_A_R currents was conspicuously decreased in CIS mice relative to those in control mice (p = 0.007, Figure 1A-B). By contrast, the phasic GABA_A_R currents, as measured by sIPSCs in PNs, did not differ between the two groups (Figure 1C-G). The parameters depicting the sIPSCs, including their amplitude (p = 0.884), frequency (p = 0.560) and dynamic properties (10-90% rise time, p = 0.698; decay constant, p = 0.458) were all similar between groups, implying that CIS exposure preferentially suppressed the tonic but not phasic GABA_A_R currents in LA. To test whether the loss of tonic inhibition was a common response evident for exposure to different types of stressors, we next measured the two forms of currents in CUS mice. The tonic currents in these mice were also weaker than those in control mice (F(2, 32) = 7.675, p = 0.002, Figure 1A-B) but similar to those in CIS mice (p = 0.388). Their sIPSCs did not differ from those in control and CIS mice (amplitude: F(2, 44) = 0.352, p = 0.705; frequency: F(2, 44) = 0.048, p = 0.953; 10-90% rise time: F(2, 44) = 0.712, p = 0.496; decay constant: F(2, 44) = 1.894, p = 0.162; n = 17, Figure 1C-G). Thus, the loss of tonic GABA_A_R currents might represent a common feature of the deficits in GABAergic signaling induced by prolonged exposure to diverse stressors. Since the tonic GABA_A_R currents in brain are activated by the ambient GABA outside of the inhibitory synapses and its content is largely limited by the activity of GABA transporter, the loss of tonic inhibition subsequent to CIS may reflect decreased GABA diffusion resulting from an enhanced GABA transporter. To test this, we employed a GABA_A_R-containing outside-out patch as a sensor of ambient GABA and compared the efficacy of GABA transport in control and CIS mice by monitoring the changes of tetanus-evoked GABA_A_R currents in response to 10 μM SKF 89976A, a GABA uptake inhibitor. We found that the SKF 89976A readily enhanced the amplitude and charge transfer of GABA_A_R current in both groups of mice and the enhancement was only slightly but insignificantly stronger in CIS mice than those in control mice (control: n = 8; CIS: n = 8; amplitude, p = 0.302; charge transfer, p = 0.171, Figure 2). Thus, it appeared that the GABA transport did not display considerable changes upon 10 days’ removal from CIS and the enduring loss of tonic inhibition in CIS mice was mainly not due to an altered GABA diffusion.

**Figure 3:**
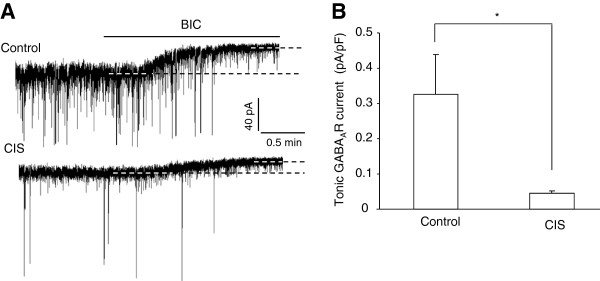
**The loss of tonic inhibition persists after 30 days of recovery from chronic immobilization stress (CIS). A**, Example traces showing the tonic GABA_A_R currents in LA PNs from control mice (upper) and mice having 30 days of recovery from CIS (bottom). **B**, Summary of the tonic GABA_A_R currents in **(A)**. * p < 0.05.

To investigate whether such loss of tonic GABA_A_R currents could last for a longer period, we continued to examine the currents in CIS mice experiencing 30 days of stress-free recovery. Compared with those in the control mice of similar age (68-75d), the tonic GABA_A_R currents in CIS mice were also weaker (control: n = 7; CIS: n = 7; p = 0.013, Figure 3). Thus, the above results demonstrated that chronic stress produced enduring loss of tonic GABA_A_R currents in the LA PNs.

**Figure 4:**
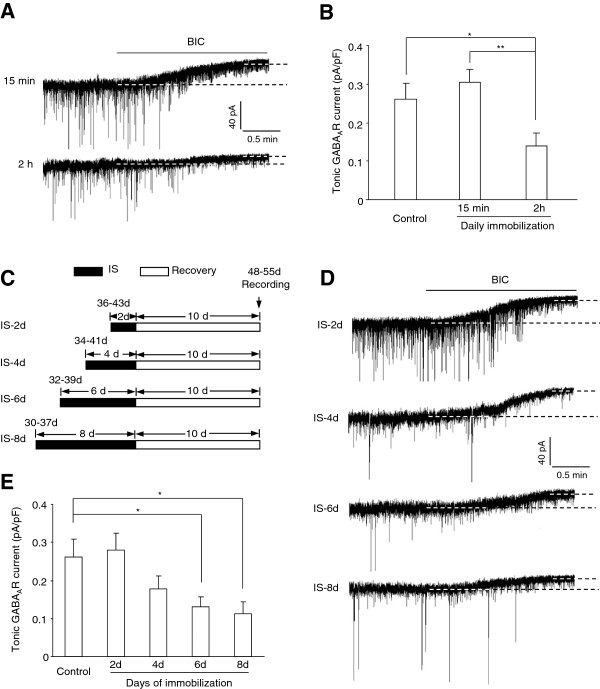
**The alteration of tonic GABA**_**A**_**R currents by chronic immobilization stress (CIS) varies with the daily immobilization duration and the total days of immobilization exposure. A**, Representative traces showing the tonic GABA_A_R currents in CIS mice subjected to 15 minutes (upper) or 2 hours (bottom) of daily immobilization. **B**, Comparisons of the tonic GABA_A_R currents between control mice and CIS mice subjected to daily immobilization with different durations. **C**, Procedures used to treat the mice with different days of immobilization stress (IS) followed by 10 consecutive days of stress-free recovery. The tonic GABA_A_R currents in the LA PNs were recorded upon the cessation of recovery. **D**, Representative traces showing the tonic GABA_A_R currents in different groups of IS mice. **E**, Comparison of the tonic GABA_A_R currents between control mice and different groups of IS mice. *p < 0.05; **p < 0.01.

The adversity of stress exposure is generally known to depend on its severity and duration of exposure. Having observed that 1 hour of daily immobilization for 10 consecutive days was sufficient to evoke loss of tonic GABA_A_R currents in LA, we asked whether the alteration of the tonic inhibition was related to the duration of daily immobilization and the total days of stress exposure. To this end, we first varied the duration of daily immobilization from 1 hour to 15 minutes or 2 hours. After 10 days recovery from CIS, the tonic GABA_A_R currents in those subjected to 2 hours daily immobilization were also dramatically decreased (n = 6, F(3, 38) = 7.895, p < 0.001, Figure 4A-B), but to a level similar to that seen in the 1 hour immobilization group (p = 0.632). By contrast, the CIS mice having daily immobilization for 15 minutes did not show noticeable changes in their tonic currents in LA (n = 9, p = 0.692 *vs* control, Figure 4A-B).

We next investigated whether the effect of stress on tonic inhibition also varied with the total days of immobilization exposure. For this, we randomly assigned the mice into different groups which were given 1 hour daily immobilization for 2, 4, 6 or 8 consecutive days respectively. 10 days after the cessation of stress exposure, the tonic GABA_A_R currents were measured in LA (Figure 4C). As depicted in Figure 4D-E, the tonic GABA_A_R currents declined progressively with the increase of exposure days and one-way ANOVA revealed a significant effect of exposure days on the tonic inhibition (F(4, 41) = 3.459, p = 0.017). Whereas 2 or 4 days of immobilization failed to have significant effect on the tonic GABA_A_R currents (IS-2d: n = 7, p = 0.806 *vs* control; IS-4d: n = 7, p = 0.256 *vs* control) in LA PNs, 6 or 8 consecutive days of immobilization markedly decreased the currents (IS-6d: n = 8, p = 0.035 *vs* control; IS-8d: n = 9, p = 0.026 *vs* control). Collectively, the above results strongly suggested that CIS-evoked loss of tonic inhibition in LA was primarily contingent on both the duration of daily immobilization and the total days of exposure.

### CORT is required for CIS-induced loss of tonic GABA_A_R currents in LA

**Figure 5:**
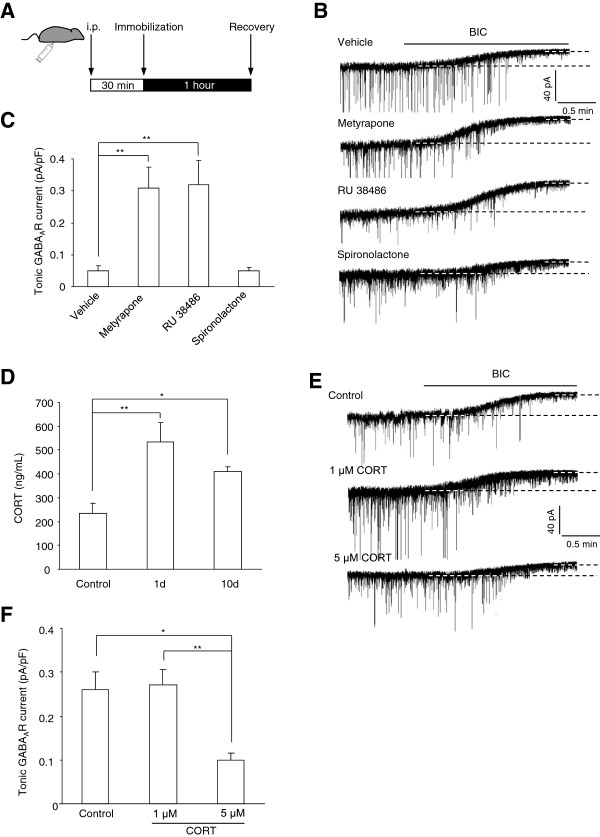
**The loss of tonic GABA**_**A**_**R currents in response to chronic immobilization stress (CIS) is mediated by corticosterone (CORT) production with subsequent activation of glucocorticoid receptor. A**, Experimental procedures used to treat CIS mice with different drugs to examine the role of CORT signaling in the altered tonic GABA_A_R currents by CIS. **B**, Example traces showing the tonic GABA_A_R currents in LA from CIS mice receiving vehicle pretreatment or pretreated with metyrapone, RU 38486 and spironolactone respectively. **C**, Summary of the tonic GABA_A_R currents in **(B)**. **D**, Comparisons of the plasma CORT levels in control mice and mice having 1 or 10 days recovery from CIS. **E**, Example traces showing the tonic GABA_A_R currents in control mice and mice fed with different levels of CORT. **F**, Summary of the tonic GABA_A_R currents in (d). *p < 0.05; **p < 0.01.

Chronic stress exposure results in the overactivation of HPA axis with a surge of CORT production, which accounts for many of its deleterious effects on the brain and behavior. CORT was also shown to be capable of modulating the expression and function of GABA_A_R [22], promoting us to speculate that excessive CORT secretion in response to CIS might mediate the decline of tonic GABA_A_R currents in amygdala. To test this, we pretreated the CIS mice with metyrapone (80 mg/kg) to block CORT synthesis 30 minutes prior to the daily immobilization (Figure 5A). Its potential effects on the altered tonic inhibition following CIS were then examined. We found that metyrapone administration thoroughly reversed the loss of tonic GABA_A_R currents (metyrapone: n = 10, F(2, 29) = 9.040, p < 0.001, Figure 5B-C) while vehicle administration had little effect (vehicle: n = 7, p = 0.084 *vs* CIS), suggesting that CORT was necessary for the loss of tonic GABA_A_R current.

**Figure 6:**
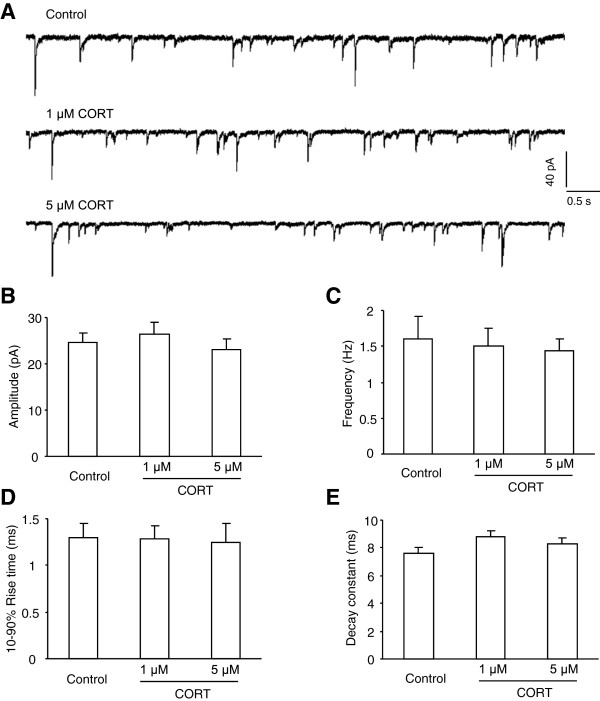
**Feeding with CORT does not affect the sIPSCs in LA PNs. A**, Representative traces showing the sIPSCs in LA PNs from control mice (upper) and mice fed with 1 μM (middle) and 5 μM CORT (bottom) for 10 consecutive days. **B**-**E**, Summary plots of the sIPSCs amplitude **(B)**, frequency **(C)**, 10-90% rise time **(D)** and decay constant **(E)** in **(A)**.

Given the critical involvement of CORT, we next asked whether the elevated CORT secretion subsequent to CIS could be maintained for a long period after stress removal. We measured the plasma CORT levels in control mice and those experiencing 1 or 10 days recovery from CIS. The results demonstrated that the CORT levels in both groups of CIS mice were far higher than that in control mice (n = 6 for each group, F(2, 15) = 7.201, p = 0.006, Figure 5D), indicating that the elevated CORT in plasma could last for at least 10 days after the cessation of CIS exposure. To explore whether CORT itself was sufficient to suppress the tonic GABA_A_R currents in LA, we fed the mice at age of 28–35 days with CORT for 10 consecutive days and the possible changes of tonic GABA_A_R currents were detected10 days after the cessation of CORT feeding. As seen in Figure 5E-F, although 1 μM CORT in drinking water failed to affect the tonic GABA_A_R currents (1 μM: n = 9, p = 0.882 *vs* control), 5 μM CORT did lead to robust decline of the currents in LA (5 μM: n = 7, F(2, 27) = 3,978, p = 0.031). Consistent with the lack of the influence of CIS on the sIPSCs, both levels of CORT did not affect the amplitude, frequency and dynamic properties of sIPSCs (amplitude: F(2, 36) = 0.512, p = 0.603; frequency: F(2, 36) = 1.025, p = 0.407; 10-90% rise time: F(2, 36) = 0.415, p = 0.663; decay constant: F(2, 36) = 2.010, p = 0.148, Figure 6). Altogether, these results highlighted a prominent role for CORT production in CIS-evoked loss of tonic inhibition in LA.

### CIS weakens tonic GABA_A_R currents through activation of glucocorticoid receptors

In brain, CORT functions through binding to GR and mineralocorticoid receptor (MR) in the target areas. To identify their roles in CIS-induced loss of tonic GABA_A_Rs currents, we pretreated the CIS mice 30 minutes prior to the daily immobilization with either RU 38486 (20 mg/kg/day), a GR antagonist or spironolactone (100 mg/kg/day), a MR antagonist (Figure 5A). While spironolactone pretreatment had no effect, pretreatment with RU 38486 effectively prevented the decline of tonic GABA_A_R currents subsequent to CIS (spironolactone: n = 7; RU 38486: n = 8, F(2, 19) = 10.538, p < 0.001, Figure 5B-C). Thus, these results implied a pivotal role of GR but not MR in the disruption of tonic inhibition mediated by CORT.

### Loss of tonic GABA_A_R current impairs GABAergic control over neuronal excitability in LA

**Figure 7:**
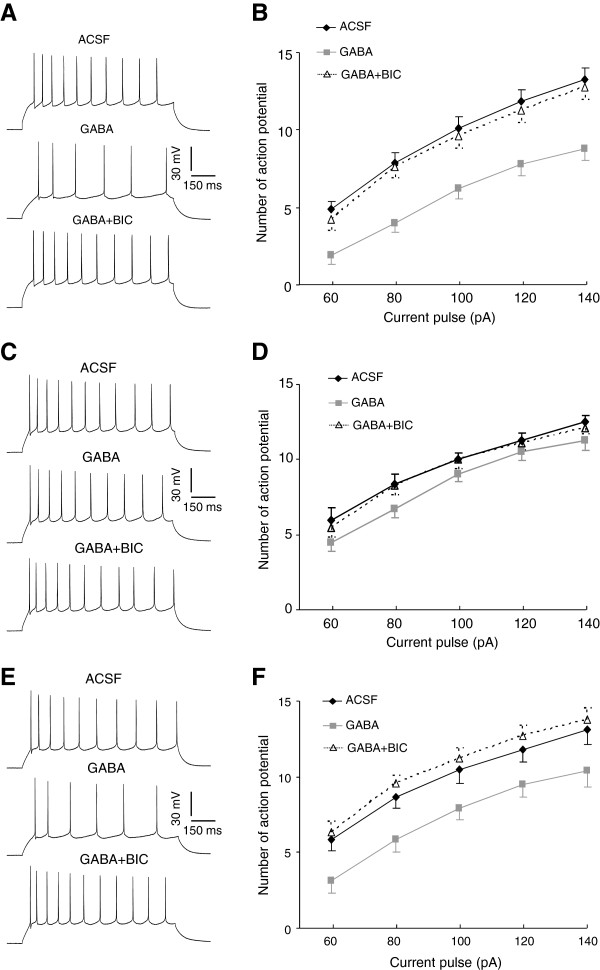
**Chronic stress leads to substantial impairment of GABAergic inhibition onto neuronal excitability in lateral amygdala (LA). A**, **B**, The GABAergic modulation of neuronal excitability in LA from control mice. **(A)** shows the firing pattern of a single LA neuron in response to a depolarizing current pulse of 100 pA when the slices are perfused with ACSF (upper), GABA (middle) and BIC (bottom) successively. **(B)** shows the number of action potential increases steadily with the increase of pulse amplitude. Bath application of GABA causes a substantial decrease of the action potential number at any given pulse amplitude, which is completely reversed by subsequent BIC application. **C**, **D**, The GABAergic modulation of neuronal excitability in LA from CIS mice. The ability of GABA to suppress the neuronal firing in CIS mice is much weaker relative to that in control mice. The other illustrations are the same as in **(A-B)**. **E**, **F**, The GABAergic modulation of neuronal excitability in LA from metyrapone-pretreated CIS mice. Metyrapone pretreatment improves the impaired GABAergic control over neuronal excitability in LA from CIS mice. The other illustrations are the same as in **(A-B)**. *p < 0.05; **p < 0.01; ***p < 0.001.

The neuronal excitability in amygdala is normally under extensive inhibitory control of GABA but undergoes substantial enhancement upon CIS. Given loss of tonic inhibition as a consequence of CIS, we speculated that such loss might impair the ability of GABA to inhibit the neuronal excitability in LA, thereby contributing to the increased responsiveness of amygdala. To test this scenario, we delivered depolarizing current pulses to the clamped neurons to evoke neuronal firing and compared the possible influence of GABA on the properties as well as the number of action potential in control and CIS mice (Figure 7A-D). The properties of action potential, including its threshold (p = 0.116), amplitude (p = 0.458) and half width (p = 0.590), did not differ between control and CIS mice and were unaltered by subsequent application of GABA (Table 1). However, GABA perfusion caused marked reduction in the spike number in both groups, which was completely reversed by subsequent application of 10 μM BIC (the main effect of treatment, control: F(2, 113) = 46.05, p < 0.001, n = 8; CIS, F(2, 113) = 9.94, n = 8, p < 0.001, Figure 7A-D). Such reduction, however, was much weaker in CIS mice relative to that in control mice (the main effect of group, F(1, 74) = 45.30, p < 0.001). Thus, the loss of tonic inhibition in response to CIS was accompanied by an impaired GABAergic suppression of neuronal firings in LA.

**Table 1:** Effect of GABA on the properties of action potential in lateral amygdala from different groups of mice

**AP parameters**	**Control (n = 8)**		***p***	**CIS (n = 8)**		***p***	**CIS + metyrapone (n = 7)**		***p***
	**ACSF**	**GABA**		**ASCF**	**GABA**		**ASCF**	**GABA**	
AP threshold (mV)	-36.29±1.28	-38.43±0.74	0.367	-40.74±2.09	-40.50±2.14	0.801	-38.97±1.21	-38.28±1.14	0.635
AP amplitude (mV)	80.25±2.41	81.45±2.50	0.597	78.83±2.05	79.57±1.51	0.595	82.73±1.45	80.60±2.98	0.386
Half AP width (ms)	2.09±0.12	2.10±0.09	0.900	2.12±0.14	2.14±0.11	0.817	2.01±0.15	2.04±0.10	0.548

AP: action potential. ACSF: artificial cerebrospinal fluid. The number in parenthesis represents the number of neurons. p values were obtained from comparisons before and after GABA application using paired *t* test.

Since our above findings have demonstrated that corticosteroid modulation was a kernel process for CIS-evoked reduction of tonic GABA_A_R currents, we hypothesized that blocking CORT synthesis might be effective in preventing the disruption of GABAergic control over neuronal excitability in CIS mice. We pretreated the CIS mice with metyrapone and examined its potential role in reversing the altered GABAergic control over neuronal excitability. The basal properties of action potential in metyrapone-treated CIS mice did not differ from those in control or CIS mice (threshold, F(2, 21) = 2.21, p = 0.099; amplitude, F(2, 21) = 1.86, p = 0.158; half width, F(2, 21) = 0.871, p = 0.482, Table 1). However, GABA caused a far greater decrease of the number of action potential in these mice relative to that in CIS mice (the main effect of group, F(1, 69) = 7.098, p = 0.009, Figure 7C-F), revealing substantial improvement in GABAergic dysfunction by metyrapone pretreatment. Together with its ability to rescue the loss of tonic GABA_A_R currents following CIS, theses results suggested that CORT production was critically engaged in the removal of inhibition in amygdala subsequent to chronic stress.

## Discussion

Here, we find that chronic exposure to either immobilization or unpredictable stress results in enduring loss of tonic GABA_A_R current in LA while leaving the phasic GABA_A_Rs unaffected. Such loss is primarily due to the production of CORT with subsequent activation of GR and leads to an impaired GABAergic control over the neuronal excitability in amygdala. Given the essential role of amygdala GABA_A_R in maintaining the appropriate expression of emotion such as fear and anxiety [9], we propose that the defective tonic inhibition may represent one of the key mechanisms through which prolonged stress exerts its persistent and detrimental action on brain’s processing of the emotionally salient events.

The GABA_A_Rs mediating the tonic and phasic inhibition in CNS differ considerably in terms of their subunit composition, subcellular localization, kinetic and pharmacological properties [23]. Despite these, they both are exquisitely sensitive to the changes in their environment. For instance, the elevated level of steroid hormone progesterone during the ovarian cycle alters the expression of both δ and γ2 subunits of GABA_A_Rs, which are the kernel subunits of GABA_A_Rs responsible for tonic and phasic inhibition respectively in many brain areas [24]. Accumulating evidence has shown that stress exposure exerts wide actions on GABA_A_Rs, ranging from changing their orthosteric and allosteric binding sites [25,26] to modulating the mRNA and protein expression of GABA_A_R subunits [26-28]. In amygdala, chronic stress has been reported to regulate the expression of several GABA_A_R subunits [29,30]. Yet, it is unknown whether these effects result in the changes of tonic and phasic inhibition in amgydala. Here, we reveal that both CIS and CUS produce enduring decline of tonic GABA_A_R currents while having negligible effects on their phasic counterparts. The loss of tonic inhibition persists even 30 days after the cessation of stress exposure, implying a striking temporal persistence of the altered tonic inhibition by chronic stress. These findings, however, do not reconcile with recent studies showing that acute immobilization stress impairs the evoked inhibitory currents in amygdala [16,31]. One possible explanation for this discrepancy is that the impaired phasic inhibition following immobilization stress is reversed after long term stress-free recovery, making it undetectable after 10 or 30 days of stress removal.

As generally known, the adversity of chronic stress is highly correlated with the severity of stressor and the duration of exposure. We find it is also the case for the altered tonic inhibition in LA. It varies dramatically with the duration of daily immobilization and the total days of immobilization stress. When the daily immobilization duration was set for 1 hour, short-term exposure (2 or 4 days) failed to affect the tonic inhibition and increasing the days of exposure (6 or 8 days) caused the reduction. On the other hand, when the immobilization exposure was set for a total of 10 days, 15 minutes of daily immobilization failed to have significant influence on the tonic inhibition, which could be readily suppressed by extending daily exposure to 1 or 2 hours. Somewhat surprisingly, the phasic GABA_A_R currents do not experience remarkable changes, suggesting a type-specific effect of chronic stress. The exact cellular mechanisms for this are not yet known. However, accumulating studies have documented that stress regulates the expression and function of GABA_A_R in a subunit- and area-specific manner. For example, chronic stress exposure decreases the expression of β1/2 subunits but has no effect on α1/2 subunits in periventricular nucleus [28]. By contrast, it increases the expression of β subunits in the hippocampus [32]. In view of the heterogeneity of the modulation of GABA_A_R by chronic stress and the marked differences between tonic and phasic inhibition, the selective reduction of tonic GABA_A_R currents may thus raise a possibility that chronic stress preferentially regulate the expression and/or function of the GABA_A_R subunits associated with tonic inhibition. It is worth noting that whereas CIS and CUS have been shown to exert distinct effects on the structural remodeling in the limbic systems [12], we find that they both impair the tonic inhibition in amygdala. We propose that it may serve as a common mechanism for the aberrant activation of amygdala in response to both paradigms of chronic stress [14,33,34].

We next observe that CORT mediates the impaired tonic inhibition in LA subsequent to chronic stress. Blocking CORT production with metyrapone during CIS effectively prevents the decline of tonic inhibition. In addition, chronic administration of CORT mimics the effects of chronic stress and results in a substantial drop of tonic GABA_A_R currents in LA. CORT administration has been shown to have a wide range of effects on GABA_A_R. It regulates the expression and function of GABA_A_R [26,34] and alters the driving force of GABA_A_R-mediated chloride currents [35]. Although the exact cellular mechanisms for these CORT actions remain undetermined, the altered GABA_A_R functionality by CORT may contribute to the loss of tonic inhibition and amygdala disinhibition subsequent to prolonged stress exposure. Such a speculation seems not to reconcile with the unaltered sensitivity of synaptic GABA_A_Rs to GABA by CORT, as shown in the current study. However, considering that synaptic versus extrasynaptic GABA_A_R differs a lot from each other in their subunit composition and dynamic modulation [36], CORT may regulate the expression and function of extrasynaptic versus synaptic GABA_A_R in different manners. Actually, these two types of GABA_A_Rs exhibit contrasting alterations under some pathological conditions. For example, the expression of synaptic GABA_A_R was increased in temporal lobe epilepsy while that of the δ subunit of GABA_A_R, a subunit located exclusively in extrasynaptic space, was decreased [37,38]. A substantial number of studies have also demonstrated that excessive CORT secretion accounts for the neuronal restructuring and the altered glutamatergic transmission in hippocampus, amygdala and prefrontal cortex [39-42], which are thought to be the primary mechanisms for the involvement of CORT in emotional and cognitive dysfunction by chronic stress [43-45]. Given the long lasting loss of tonic inhibition in response to chronic stress or CORT administration, we speculate it may provide an alternative mechanism through which CORT mediates the persistent deleterious effects of chronic stress.

In the brain, CORT functions primarily through GR and MR. While both receptors are colocalized in amygdala [46], we observe that it is GR rather than MR that mediates the disruption of tonic inhibition subsequent to chronic stress. This is likely to be associated with their distinct pharmacological properties [47]. The CORT affinity of MR is ten times higher than that of GR [48]. The high CORT affinity makes MR to be heavily occupied by basal level of CORT. By contrast, GR is heavily occupied only when the circulating CORT is elevated under conditions such as stress exposure, which renders GR ideal for mediating the biological function of stress. Consistent with the pivotal role of GR in the diminished tonic inhibition, a wealth of data has also documented the involvement of GR in the dysregulation of HPA axis and memory deficits following chronic stress [49,50] and antagonism of GR signaling has been proposed as a therapeutic target in stress-related psychiatric diseases [51,52].

Lastly, we find that along with the long lasting loss of tonic inhibition, the ability of GABA to suppress neuronal firing is greatly impaired subsequent to chronic stress. Since the phasic GABA_A_R currents do not experience considerable changes, this GABAergic dysfunction is most likely due to the loss of tonic inhibition. It has been shown that the charge carried by the activation of tonically active GABA_A_Rs is three to five times larger than that carried by phasic inhibition [17,19]. Thus, it is not surprising that the deficit of tonic inhibition is sufficient to lead to the disruption of GABAergic control over neuronal excitability. Recently, a few studies have implicated the defective tonic inhibitory tone in amygdala in the pathogenesis of some neuropsychiatric disorders [53,54]. Further studies are needed to uncover its functional role in the adverse effects of chronic stress on the brain and behavior.

## Conclusions

We have shown in this study that chronic stress exposure triggers enduring loss of tonic but not phasic GABA_A_R currents in amygdala which is dependent on stress-evoked CORT production with subsequent GR activation. We conclude that the loss of tonic inhibition contributes to amygdala disinhibition following chronic stress and may thus account for the development of neuropsychiatric disorders.

## Methods

### Animals

Male C57BL/6 J mice were subjected to chronic stress exposure at age of 28–35 days except for those stated in Figure 3C. All animals were housed in groups of 3–5 with *ad libitum* access to food and water unless specified in stressed mice and maintained in a temperature and humidity controlled room with a light/dark cycle of 12 hours. All experimental procedures followed the guidelines of National Institutes of Health and were approved by the ethics committee of Nanchang University.

### Models of chronic stress

Chronic immobilization stress (CIS) and chronic unpredictable stress (CUS) were employed in the present experiment. Mice assigned to CIS were placed in a restraint cylinder at around 2 pm for 1 hour per session, one session per day and for 10 consecutive days, unless stated otherwise. For the CUS paradigm, the mice were given 2 stressors per day for 10 consecutive days. The stressors applied were randomly selected from 8 stressors and thus unpredictable for the mice. The 8 stressors included forced swim for 4 min, lights on overnight, lights off for 3 hr during the light period of the light/dark cycle, cold room exposure (temperature was set at 10°C) for 1 h, food and water deprivation overnight, gentle cage shaking for 1 h, immobilization for 1 h and wet bedding overnight. The control mice were transferred in their home cages to the experimental room, gently handled for 2–4 minutes and returned back to the feeding room about 1 hour later.

### Corticosterone assay

Animals were anesthetized with ether at around 1-2 pm and the blood was collected through cardiac puncture into heparinized tubes. Samples were centrifuged at 3000 rpm for 20 minutes at 4°C. Sera were collected and stored at -80°C until assayed. Plasma CORT was measured by specific radioimmunoassay with ELISA kit (Abcam). To avoid the potential inter-assay variation, all samples were measured in the same assay. The standard curve (1–100 ng/mL) and samples were run in triplicate.

### Drugs

CORT was freshly prepared in the drinking water and delivered in opaque bottles to protect it from light. The stock solution of RU 38486, spironolactone and metyrapone were made using EtOH (<0.1% at final concentration) and administered intraperitoneally 30 minutes prior to the daily immobilization. CORT was purchased from Sigma-Aldrich and the others were from Tocris Bioscience.

### Electrophysiology

Amygdala slices were prepared as previously described [55]. Briefly, mice were sacrificed by decapitation and brains were quickly removed to ice-cold oxygenated (95% O_2_/5% CO_2_) artificial cerebrospinal fluid (ACSF) containing (in mM): 124 NaCl, 2.5 KCl, 1 MgSO_4_, 2.5 CaCl_2_, 10 glucose, and 26 NaHCO_3_ (pH = 7.30). Slices containing lateral amygdala (LA) of about 350 μm were cut with a Leica VT 1000S tissue slicer and maintained at room-temperature for at least one hour before recording. Slices were transferred to a recording chamber continuously superfused with ACSF at a constant rate of about 60 ml/h. The whole-cell patch clamp was made in the projection neurons (PNs) of LA with an Axon 700B amplifier. The patch pipettes for recording GABAergic currents were filled with (in mM): 100 CsCl, 30 Cs-methanesulfonate, 5 NaCl, 2 MgCl_2_, 10 HEPES, and 0.2 EGTA, 2 ATP-Na, 0.1 GTP-Na. The pH was adjusted to 7.3 with CsOH and osmolarity to 285 mOsm with sucrose. In experiments where action potentials were evoked, CsCl and Cs-methanesulfonate were replaced by equal concentrations of Kgluconate. To record the phasic and tonic GABA_A_R currents, 20 μM APV, 20 μM DNQX and 5 μM CGP 52432 were routinely added into the bath solution to block the ionotropic glutamate receptors and B type GABA receptors. In experiments where tonic GABA_A_R currents were recorded, 20 μM GABA was included in the ACSF to ensure the activation of extrasynaptic GABA_A_Rs. The spontaneous inhibitory postsynaptic currents (sIPSCs) were collected 2–3 minutes prior to GABA application. To evoke action potentials in the PNs, cells were recorded at current clamp mode and the depolarizing current pulses of increasing amplitude were delivered. To measure GABA diffusion in amygdala slice from control and CIS mice, we performed an outside-out patch containing GABA_A_R and inserted this patch into slice [55]. The GABA_A_R currents in response to the diffused GABA following a short-lasting tetanus delivered to LA (4 stimuli, 100 Hz) were recorded and the effects of GABA uptake inhibitor were monitored. The pipette resistance was 3–7 MΩ. The membrane potential was held at -70 mV and the junction potential of about 12 mV were uncorrected. Series resistance (Rs) was in the range of 10–20 MΩ and monitored throughout the experiments. If Rs changed more than 20% during recording, the data were not included in analysis.

### Data analysis and statistics

Data were low-pass filtered (3KHz) and digitized at 10 KHz. Tonic GABA_A_R currents were defined as the currents blocked by bicuculline (BIC) and measured as previously described [56]. The amplitude, frequency and dynamic parameters of sIPSCs were analyzed offline using MiniAnalysis (*Synaptosoft, Inc*). The spike threshold was identified at the point where the action potential was initiated and showed a >10 fold change in the rising rate. The amplitude of AP was measured as the voltage difference between the threshold and the peak of the action potential. The half AP width was measured at half-height between the threshold and the peak of the action potential. All data were expressed as Means±SEM. The comparisons of the intensity of tonic inhibitory currents and the parameters depicting sIPSCs or action potential were obtained by using ANOVAs followed by *post hoc t* test. Statistical significances were considered at p < 0.05.

## Abbreviations

LA: Lateral amygdala; PN: Projection neuron; GABAAR: GABAA receptor; CIS: Chronic immobilization stress; CUS: Chronic unpredictable stress; sIPSC: Spontaneous inhibitory postsynaptic currents; CORT: Corticosterone; GR: Glucocorticoid receptor; MR: Mineralocorticoid receptor.

## Competing interests

The authors declare that they have no competing interests.

## Authors’ contribution

LZ and WM performed most of the electrophysiological experiments and analyzed the data. SC and HY made the mice models of chronic immobilization and unpredictable stress. PH and CW measured the neuronal excitability in different groups of mice. XX injected the mice with different drugs. PB conceived and designed the project. PB and PW wrote the manuscript. All authors read and approved the final manuscript.
